# Suicidality in adolescents with Complex Regional Pain Syndrome (CRPS)

**DOI:** 10.1192/j.eurpsy.2023.1287

**Published:** 2023-07-19

**Authors:** S. Moustakil, P. Guillaume, A. Letessier-Selvon

**Affiliations:** Child and adolescent Psychiatry Department, Pyrenees Hospital Center, Pau, France

## Abstract

**Introduction:**

Complex regional pain syndrome (CRPS) is a rare condition associated with chronic pain. It is an inflammatory and neuropathic disorder principally characterized by involvement of the autonomic nervous system. The etiology of the syndrome is not clear and the known treatment modality is also very complicated.

**Objectives:**

Extant literature has shown the relationship between CRPS and suicidal behaviours in adults but less data are available in adolescents. This literature review aims to synthesize and evaluate the existing studies assessing suicidality in CRPS adolescents.

**Methods:**

A narrative review of the literature focusing on CRPS and chronic pain in adolescents and their associations with suicidal behavior including suicidal ideations, suicide attempts and death by suicide.

**Results:**

The studies of suicidality factors in adolescents evaluated chronic pain in general. Those who studied CRPS specifically did not look for its association with suicide risk. In fact, adolescents who suffer from chronic pain present increased risk for suicide ideations and suicidal attempts. Furthermore, no available data have demonstrated the association between chronic pain and suicide. Additionally, among adolescents with CRPS, the risks of somatization, anxiety, and depression are higher. The duration of pain, depression and eating disorders has been shown to be associated with increased suicidality.

**Conclusions:**

Our findings suggest that CRPS is associated with higher risks for suicidal ideation, suicidal attempts compared to the general population. The risk factors underlying suicidal behavior in CRPS patients are not studied enough and require further investigation.

**Disclosure of Interest:**

None Declared

